# Gut microbiota composition is associated with environmental landscape in honey bees

**DOI:** 10.1002/ece3.3597

**Published:** 2017-11-30

**Authors:** Julia C Jones, Carmelo Fruciano, Falk Hildebrand, Hasan Al Toufalilia, Nicholas J Balfour, Peer Bork, Philipp Engel, Francis LW Ratnieks, William OH Hughes

**Affiliations:** ^1^ School of Life Sciences University of Sussex Brighton UK; ^2^ School of Earth Environment and Biological Sciences Queensland University of Technology Brisbane QLD Australia; ^3^ European Molecular Biology Laboratory, Structural and Computational Biology Unit Heidelberg Germany; ^4^ Max Delbrück Centre for Molecular Medicine Berlin Germany; ^5^ Department of Bioinformatics University of Würzburg Würzburg Germany; ^6^ Department of Fundamental Microbiology University of Lausanne Lausanne Switzerland

**Keywords:** amplicon sequencing, bacterial microbiota, honey bee, landscape exposure, oilseed rape

## Abstract

There is growing recognition that the gut microbial community regulates a wide variety of important functions in its animal hosts, including host health. However, the complex interactions between gut microbes and environment are still unclear. Honey bees are ecologically and economically important pollinators that host a core gut microbial community that is thought to be constant across populations. Here, we examined whether the composition of the gut microbial community of honey bees is affected by the environmental landscape the bees are exposed to. We placed honey bee colonies reared under identical conditions in two main landscape types for 6 weeks: either oilseed rape farmland or agricultural farmland distant to fields of flowering oilseed rape. The gut bacterial communities of adult bees from the colonies were then characterized and compared based on amplicon sequencing of the 16S rRNA gene. While previous studies have delineated a characteristic core set of bacteria inhabiting the honey bee gut, our results suggest that the broad environment that bees are exposed to has some influence on the relative abundance of some members of that microbial community. This includes known dominant taxa thought to have functions in nutrition and health. Our results provide evidence for an influence of landscape exposure on honey bee microbial community and highlight the potential effect of exposure to different environmental parameters, such as forage type and neonicotinoid pesticides, on key honey bee gut bacteria. This work emphasizes the complexity of the relationship between the host, its gut bacteria, and the environment and identifies target microbial taxa for functional analyses.

## 1 INTRODUCTION

Individual animals are often considered discrete entities; however, the microbial symbionts they host are increasingly recognized as key components in their evolutionary and ecological success (Bosch & McFall‐Ngai, 2011; Brucker & Bordenstein, 2012; Franchini, Fruciano, Frickey, Jones, & Meyer, 2014; Gibson & Hunter, 2010; Hildebrand et al., 2012; Moran, McCutcheon, & Nakabachi, 2008; Moya, Peretó, Gil, & Latorre, 2008). Insects harbor bacteria with diverse roles ranging from nutrition to defense, and with influences on reproduction and speciation (e.g. Brucker & Bordenstein, 2013; Feldhaar, 2011; Jaenike, Unckless, Cockburn, Boelio, & Perlman, 2010). Many of these symbionts are part of the gut bacterial community and in social insects in general, the gut microbial community has been reported to be associated with a range of traits including invasive behaviors, nest sanitation, longevity, fecundity, and health (Cox‐Foster et al., 2007; Engel et al., 2016; Ishak et al., 2011; Martinson et al., 2011; Rosengaus, Zecher, Schultheis, Brucker, & Bordenstein, 2011).

Social insects, and specifically honey bees, are important models for further determining the extraordinary range of influences of microbial communities on their hosts. Recent honey bee colony losses worldwide call for a more in‐depth understanding of the pathogenic and mutualistic components of the microbial communities of this ecologically and economically important pollinator (Gallai, Salles, Settele, & Vaissière, 2009; Ollerton, Winfree, & Tarrant, 2011), and specifically the association between the environment and microbial community (Engel et al., 2016). Importantly, the ecological and evolutionary processes shaping the microbial community and host associations, ranging along a spectrum from tightly coevolved obligate relationships to facultative relationships, are as yet not well understood.

Losses of honey bees and other pollinators are thought to be due to exposure to multiple interacting stressors, including disease, pesticide exposure, flower availability, and the importation of nonnative bees (Goulson, Nicholls, Botías, & Rotheray, 2015). One factor that is likely shaped by these different stressors, and is critical to the health and success of colonies, is the composition and function of their microbial community (e.g. Engel, Martinson, & Moran, 2012; Koch & Schmid‐Hempel, 2011b; Moran, 2015). In recent surveys, adult honey bees and bumblebees have been shown to harbor a relatively simple and unique gut microbiota that is not present in solitary bees (Cox‐Foster et al., 2007; Jeyaprakash, Hoy, & Allsopp, 2003; Koch & Schmid‐Hempel, 2011a; Martinson et al., 2011; Mohr & Tebbe, 2006). Sociality has therefore been suggested to facilitate the vertical transmission of gut bacteria and allow for the coevolution of the host and gut bacteria that may be critical to bee health (Koch & Schmid‐Hempel, 2011b; Kwong et al., 2017; Martinson et al., 2011; Moran, 2015; Olofsson & Vásquez, 2008). Genomic and metagenomic analyses suggest that different taxa within this core microbial community are likely involved in different functions (Ellegaard et al., 2015; Engel, Bartlett, & Moran, 2015; Engel, Stepanauskas, & Moran, 2014; Engel et al., 2012; Kwong, Engel, Koch, & Moran, 2014; Lee, Rusch, Stewart, Mattila, & Newton, 2015), and therefore host exposure to different ecological pressures may select for flexibility in the abundance of the different gut microbial taxa. Specifically, a combination of 16S rRNA community surveys and metagenomics studies has shown that the gut community of worker honey bees is dominated by nine bacterial species clusters that make up 95%–98% of the community (Babendreier, Joller, Romeis, Bigler, & Widmer, 2006; Corby‐Harris, Maes, & Anderson, 2014; Jeyaprakash et al., 2003; Martinson et al., 2011; Moran, Hansen, Powell, & Sabree, 2012; Sabree, Hansen, & Moran, 2012). These include five core species clusters, two abundant and ubiquitous gram‐negative species clusters from the Proteobacteria phylum, *Snodgrasssella alvi* and *Gilliamella apicola* (Kwong & Moran, 2013), two abundant and ubiquitous gram‐positive species clusters in the Firmicutes Phylum referred to as *Lactobacillus* Firm‐4, and *Lactobacillus* Firm‐5 clades (Babendreier et al., 2006; Martinson et al., 2011), and the species cluster *Bifidobacterium asteroides* from the Actinobacterium phylum (Bottacini et al., 2012; Scardovi & Trovatelli, 1969). Four additional species clusters that are prevalent but can occur at lower frequencies are the proteobacteria – *Frischella perrara*,* Bartonella apis*, and two Acetobacteraceae, Alpha2.1, and Alpha 2.2 (*Parasaccharibacter apium*) (Corby‐Harris, Synder, et al., 2014; Engel, Kwong, & Moran, 2013; Kešnerová, Moritz, & Engel, 2016; Martinson et al., 2011). These four species clusters have been found to be either restricted in their niches in the bee gut, or are more generalists that are also found in the hive environment, as in Alpha 2.2 in particular (Corby‐Harris, Synder, et al., 2014; Kwong & Moran, 2016).

Importantly, the current paradigm is that the core bacterial community of honey bees is relatively constant across populations and geographical areas (Cox‐Foster et al., 2007; Jeyaprakash et al., 2003; Martinson et al., 2011; Mohr & Tebbe, 2006; Moran et al., 2012; Sabree et al., 2012). Here, we test this by comparing the gut microbial communities of honey bees in two landscapes using 16S rRNA gene profiling. We focus on exposure to the mass‐flowering crop oilseed rape (OSR, also known as canola). OSR is one of the most important crops worldwide occupying 3% of the land area in the United Kingdom (DEFRA 2013), and often dominating the local landscape. It has also been a focus of recent debate over the application of neonicotinoid pesticides that have been implicated in pollinator declines (Suryanarayanan, 2015). We therefore compared the gut bacterial communities of honey bees exposed to OSR farms with those from agricultural environments distant to fields of flowering OSR.

## 2 MATERIALS AND METHODS

### 2.1 Sites and sampling

**Figure 1:**
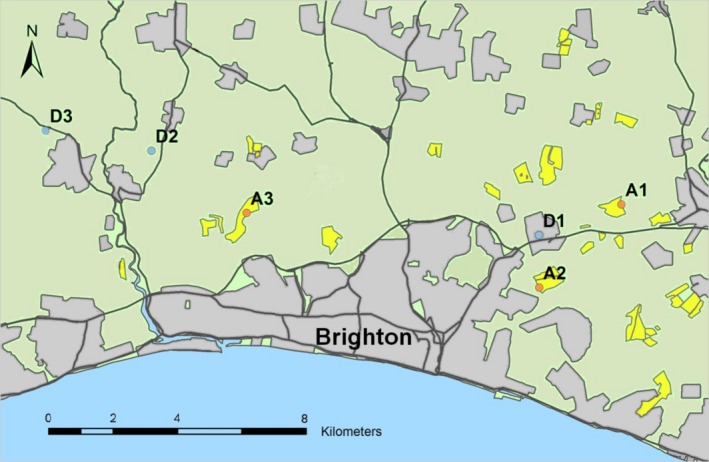
Apiary locations in two different landscape types in Sussex, United Kingdom. Oilseed rape (OSR) fields are highlighted in yellow. Apiaries are labelled and colored by their landscape exposure type (orange, OSR farmland; blue, areas distant from OSR farmland)

Thirty‐six honey bee colonies were maintained using standard beekeeping methods by the same beekeeper at the University of Sussex for 1 year prior to the experiment. As detailed in Balfour et al. (2017), colonies were equalized on 31 March and 1 April 2014 during unfavorable foraging conditions to ensure that the vast majority of foragers were within the hive and worker population could be assessed. Each colony had a marked laying queen, four frames of brood, six frames of adult worker bees, two–three frames of honey, 0.5–1 frames of pollen, and two frames of empty wax foundation comb. Visual inspection suggested all colonies were disease free. Colonies differed in genetic background but were randomly allocated to landscapes so differences in genetic background would not confound results. The honey bee colonies were then placed at six different locations in two landscape types in the southern UK (on 2–4 April 2014; Figure 1): (i) farmland areas immediately adjacent to (<5 m) large (≥0.38 km^2^) oilseed rape (OSR) fields that were in flower and had been seed‐treated with thiamethoxam (Cruiser, Syngenta Ltd.); (ii) agricultural land distant to OSR (Distant) with the nearest OSR field boundaries being located ≥1.25 km from hives, and therefore little visited as average foraging distances are short, <1.1 km, during the OSR blooming period (April–May) (Couvillon, Schürch, & Ratnieks, 2014). All study sites were selected to be as similar as possible in other landscape factors, including elevation, soil type, exposure, and land use. Information on pesticide usage was supplied by local agronomists and farm owners. Adult forager bees found on the exterior of the colony were sampled from each apiary just after peak OSR flowering time when workers at the OSR farms had been foraging and storing nectar and pollen from the OSR. Adult forager bee samples were immediately frozen and stored for extraction and sequencing after collection (each of the samples analyses is a pool of three bees per colony, see Table S1 for sampling details). Pollen pellets, collected from returning foragers during early and full OSR bloom using a trap fitted to each hive, were identified to determine the average amount of OSR pollen per apiary. This was conducted for both landscape categories. The average neonicotinoid residues (thiamethoxam + clothianidin) were quantified in pollen and honey samples for both landscape types in order to determine residues in each landscape (OSR; Distant). Stored honey samples were taken from each hive on May 15, near the end of OSR bloom to reflect foraging during the bloom, and pooled across colonies per apiary. Specifically, sealed honey was collected from multiple previously empty frames and locations within each colony to provide a representative sample from the OSR bloom period.

As outlined in Balfour et al. (2017), samples were analyzed for neonicotinoid concentrations (thiamethoxam and its metabolite clothianidin) by SAL (Scientific Analysis Laboratory Ltd., Cambridge), an accredited (UK Accreditation Service) contract analytical laboratory. SAL's extraction method is based on the QuEChERS extraction technique which uses water and acidified acetonitrile as an extraction solvent (Kamel, 2010). Magnesium sulfate and ammonium acetate (as a buffer) were added to induce solvent partitioning. Quantitation was assessed against a series of known calibration standards dissolved in a methanol:water solution. Deuterated clothianidin (Clothianidin‐d3) was used as an internal standard preextraction, to correct for losses during extraction and to compensate for matrix effects (suppression or enhancement) during analysis. The limit of quantification (LOQ) and detection (LOD) were 0.1 μg/kg for both thiamethoxam and clothianidin and for both pollen and honey.

### 2.2 DNA extraction, amplification, and sequencing

After thawing for 1–2 min, the gut of each individual, from the midgut to the hindgut, and not including the crop, was dissected under sterile conditions. DNA extractions of individual guts were performed immediately after dissection using the Zymo Research Tissue and Insect DNA MiniPrep (Cambridge Biosciences, Cambridge, UK) following the manufacturer's protocol. Illumina libraries were prepared following the method outlined by Caporaso et al., 2012 (Caporaso et al., 2012). Briefly, the bacterial V4 region of the 16S ribosomal gene was amplified from each DNA template in triplicate using the universal primers 515F and 806R tailed with Illumina barcoded adapters using the PCR conditions 95°C for 10 min, 35 cycles of 95°C for 30 s, 59°C for 30 s, 72°C for 1 min, and a final extension of 72°C for 7 min. PCR products were sent to the Plateforme d'Analyses Génomiques of the Institut de Biologie Intégrative et des Systèmes (IBIS, Université Laval, Quebec City, Canada, http://www.ibis.ulaval.ca/?pg=sequencage). The amplicons were purified using the Axygen Axyprep Mag PCR clean‐up kit (Corning). The quality of the products was assessed using a DNA7500 chip on a Bioanalyser 2100 (Agilent Technologies), quantified using a nanodrop (Thermo Scientific) and then pooled in an equimolar ratio. The quality of the final amplicon pool was rechecked as previously described, quantified using Quant‐iT picogreen ds DNA Assay (Thermo Scientific) and sequenced on an Illumina MiSeq (Illumina) using a v3 600 cycle kit. All sequences have been deposited in NCBI's Sequence Read Archive (SRA PRJEB23223).

### 2.3 Sequence processing and characterization of microbial communities

The LotuS pipeline was used for amplicon sequence processing (Hildebrand, Tadeo, Voigt, Bork, & Raes, 2014) using the following optional LotuS command line options: “‐p miSeq derepMin 8:1,4:2,3:3 –simBasedTaxo 2 –refDB SLV thr 8.” The pipeline was used to demultiplex reads with modified quality filtering to accommodate for the increased MiSeq sequence length, trimming reads to 220 bp, and rejecting reads with an accumulated error <1, requiring unique reads to be present at least eight times in one sample, four times in two, or three times in three separate samples.

In total, 11,636,723 reads were clustered at sequence level with UPARSE (Edgar, 2013), creating a set of de novo OTUs that can later be compared to databases of known sequences. Chimeric OTUs were removed against a specialized database of high‐quality reference sequences (http://drive5.com/uchime/rdp_gold.fa) using uchime (Edgar, Haas, Clemente, Quince, & Knight, 2011). High‐quality paired seed sequences were subsequently extracted for each OTU, merged with FLASH (Magoč & Salzberg, 2011), and aligned with Lambda (Hauswedell, Singer, & Reinert, 2014) against a custom 16S rRNA gene database that included representatives of all major known bacterial taxa associated with honey bees (developed by P. Engel, publicly available online on the LotuS website). Additionally, all sequences were aligned against the Greengenes and Silva SSU databases using Lambda (Hauswedell et al., 2014) as well as classified with RDP classifier (Wang, Garrity, & Tiedje, 2007) in order to detect and exclude any chloroplast or mitochondrial sequences in downstream analyses. The LotuS least common ancestor algorithm was used to assign a taxonomic identity based on the alignments to known bee taxa. OTUs were summed to genus, family, class, and phylum level per sample, according to their taxonomic classification.

### 2.4 Statistical analyses and comparisons of microbial communities

All analyses, unless otherwise specified, were conducted using the LotuS outputs in R with the packages *vegan*,* phyloseq*,* phangorn,* and *ggplot2* (Castro‐Conde & de Uña Álvarez, 2014; McMurdie & Holmes, 2013; Oksanen et al., 2016; Paradis, Claude, & Strimmer, 2004; Schliep, 2011; Wickham, 2009). To reduce errors in estimation and false positives due to different numbers of sequences per individual, samples were rarefied to the smallest number of sequences per individual observed. To test for the consistency of the rarefaction, samples for each dataset were rarefied five times. For each of the rarefied matrices, pairwise sample dissimilarity matrices (Bray‐Curtis, UniFrac distances) among individuals were computed. Finally, the dissimilarity matrix obtained from the first rarefied dataset was compared with each of the dissimilarity matrices obtained from the other rarefied datasets by computing their correlation and testing its significance with a Mantel test (Mantel, 1967). Both the exploratory analyses and the tests of hypotheses described below were also performed on all the rarefied samples and inspected for consistency. Comparisons between rarefied samples using pairwise distances were found to be globally concordant (correlation 0.69–1; Mantel test significant in all cases). The results of the analyses were also always consistent across different rarefactions. For these reasons, only the results based on the first rarefied sample will be presented here.

To investigate patterns of microbial community diversity, we computed dissimilarity matrices using Bray–Curtis dissimilarity and Unifrac weighted and unweighted distances. Bray–Curtis dissimilarity reflects community composition, while UniFrac distances take into account the phylogenetic relationships among members of the bacterial communities (Lozupone & Knight, 2005). UniFrac distances are then either weighted by OTU abundance or unweighted, where only the presence/absence of taxa/OTUs is considered. These dissimilarity matrices were used to produce exploratory ordinations using nonmetric multidimensional scaling (nMDS) (Kruskal, 1964a,1964b). Hypothesis testing was carried out using permutational MANOVA (PERMANOVA) (Anderson, 2001). This approach is analogous to multivariate analysis of variance (MANOVA) but uses a dissimilarity matrix as the dependent variable (as opposed to a set of continuous variables as in MANOVA). Being analogous to a MANOVA, in PERMANOVA variation in distances is partitioned in terms (two factors – landscape type and site in our case, with site nested in landscape type) and tested for significance using a permutational procedure (1,000 permutations). In addition, we calculated the Shannon diversity index, a commonly used metric where both taxon richness and evenness of OTUs in each sample is accounted for, for each individual with the “diversity” function in *vegan* and tested for differences between groups using ANOVA.

To identify variation in bacterial taxa in honey bees exposed to different landscapes, we used the raw counts of the number of sequences that were assigned to the different OTUs. To test which OTUs were differentially represented between the two groups, we used two different procedures (Weiss et al., 2017). First, we used the procedure suggested by McMurdie & Holmes, (2014) on a dataset of nonrarefied samples where taxa with <500 reads were excluded. This procedure overcomes the need for rarefaction and uses the method implemented in the software DESeq2 (Love, Huber, & Anders, 2014), which is normally used to detect differential gene expression in RNAseq data. The DESeq2 method fits a model based on negative binomial distribution to test for differences in gene expression (in this case read counts) between two a priori defined groups. We then controlled for false discovery rate using the Benjamini and Hochberg procedure (Benjamini & Hochberg, 1995). It has recently been shown that the procedure based on DESeq2 has the advantage of increased sensitivity on smaller datasets (<20 samples per group) but tends toward a higher false discovery rate with more samples, very uneven (>10×) library sizes and or compositional effects (Weiss et al., 2017). Because of these potential limitations, we also used the analysis of composition of microbiomes (ANCOM) (Mandal, Van Treuren, & White, 2015). This procedure has recently been found to appropriately control for false discovery rate (Weiss et al., 2017). ANCOM compares the log ratio of the abundance of each taxon to the abundance of all the remaining taxa one at a time, and the Mann–Whitney U test is then calculated on each log ratio (Mandal et al., 2015; Weiss et al., 2017). Here, we used the R implementation of the procedure (version 1.1‐3).

## 3 RESULTS

### 3.1 Bacterial sequences and classification

**Figure 2:**
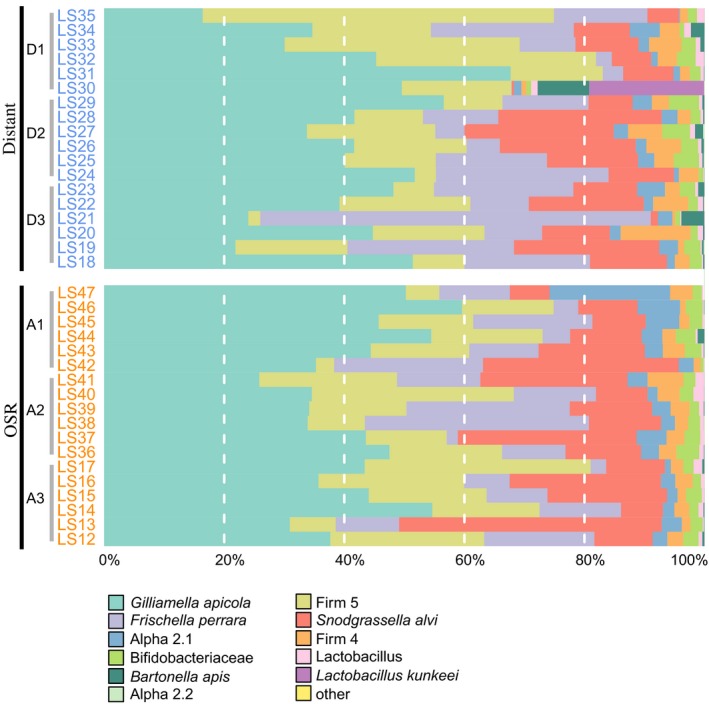
Taxonomic composition of the gut microbiome of honey bees exposed to different landscapes. The proportion of each taxa in the total microbiome is represented as the proportion of the colored bar. OSR, oilseed rape (OSR) farmland; Distant, areas distant from OSR farms. Individual apiaries are indicated with a gray bar and label

We obtained a total of 11,636,723 16S rRNA V4 region sequences from the 108 sampled bees from the two landscape exposure conditions. After quality filtering, the number of sequences obtained per sample ranged from 236,463 to 400,075 reads which clustered in a total of 449 different OTUs. Unsurprisingly, the major bacterial taxa previously found to dominate the gut community of honey bees were represented in high proportions in the samples studied here (Figure 2). Using a custom honey bee bacterial database of currently available genomes of bee gut bacteria, we were able to assign 92% of the sequence reads to species level (99.93% to phylum level, 98% to family, and 95% to genus level) and verify that the major previously identified taxa or strains were present in our data (Neisseriaceae, *S. alvi*; Orbaceae, *G. apicola* and *F. perrara*; Lactobacillaceae, Firm‐4 and Firm‐5 species groups (genus *Lactobacillus*) and *Lactobacillus kunkeei*); *Bifidobacteriaceae*; Rhizobiales, Bartonellaceae (Alpha 1; including *B. apis*); Acetobacteraceae (Alpha 2.1 and 2.2), see also (Moran, 2015) and Figure 2).

### 3.2 Landscape exposure and microbiomes

In honey bee colonies placed on OSR farms, 49% of the pollen collected was oilseed rape. Colonies located distant from OSR farms collected significantly less oilseed rape pollen than colonies adjacent to OSR farms (9%; ANOVA, *F*_1,5_ = 14.1, *p *=* *.020; see also Balfour et al., 2017). Further, pollen analysis also showed that the main alternative forage source across all six study sites were *Prunus spinosa* (~15%) and *Salix* spp. (~15%). Other less common species (<5% across study sites) included as follows: *Allium ursinum* (site D3), *Crocus* spp. (A1), *Endymion nonscriptus* (A1, D1, D2), *Taraxacum officinale* (ubiquitous), *Malus domestica* (A2), *Pyrus communis* (A1), and *Vicia faba* (A3, D2). The average neonicotinoid residues (thiamethoxam + clothianidin) of the stored pollen and honey samples in the colonies during OSR bloom were on the low side of the range previously reported (Botías et al., 2015; Cutler & Scott‐Dupree, 2014; Pilling, Campbell, Coulson, Ruddle, & Tornier, 2013; Rolke, Fuchs, Grünewald, Gao, & Blenau, 2016; Thompson & Harrington, 2013). The average residues from colonies maintained on OSR farms was 0.76 ppb, significantly greater than residues found at apiaries distant to OSR farms where the majority of samples collected were below detection levels (<0.1 ppb) with an average of 0.21 ppb (ANOVA, *F*_1,5_ = 8.1, *p *=* *.048; see also Balfour et al., 2017).

**Figure 3:**
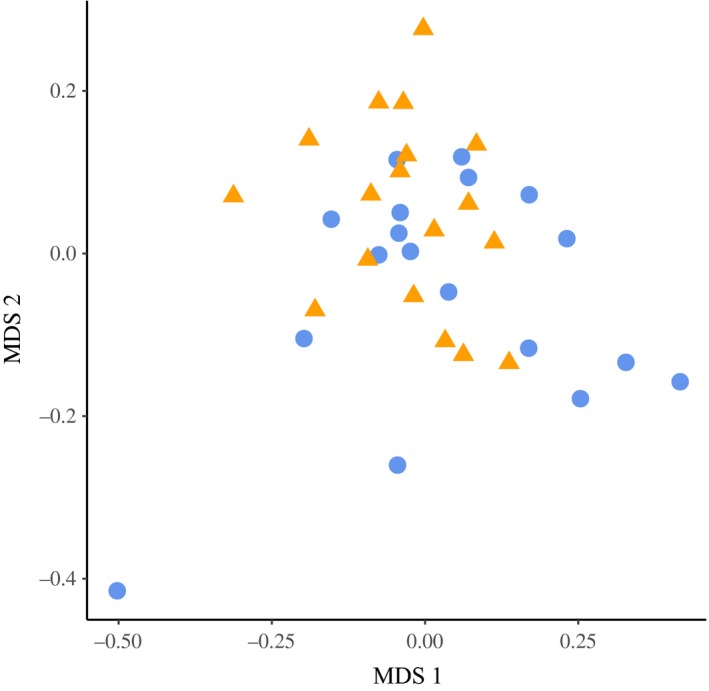
Nonmetric multidimensional scaling plot (based on Bray–Curtis distances) of OTU frequency for the gut microbial communities of honey bees in oilseed rape farmland (triangles) or farmland distant from oilseed rape (circles)

Honey bees from the two landscape types showed significant differences in their gut microbial communities using two comparisons (PERMANOVA: *p* = .004 using Bray–Curtis dissimilarity indices, *p* = .042 using unweighted UniFrac distances, Table 1), the PERMANOVA comparison using weighted UniFrac distances was not significant (*p* = .642). We also find substantial – and significant – variation among sites and a substantial proportion of residual variance (Table 1). In fact differences between landscape types accounted for 1%–6% of total variance, depending on the dissimilarity used, while differences between individual sites accounted for a higher percentage of the total variance (17%–27%; Table 1). The nonmetric multidimensional scaling (nMDS) plot shows a degree of separation, but also overlap, in the microbial communities of bees exposed to OSR farms and regions distant from OSR farms (Figure 3). Gut microbiome diversity was not significantly different in bees exposed to the different landscape types (ANOVA: *F*_1,34_ = 0.07, *p* = .79).

**Table 1:** Comparison of variation in taxa/OTUs diversity among different landscape types and sites (as a factor nested in landscape type; PERMANOVA based on Bray–Curtis dissimilarity indices and UniFrac weighted and unweighted distances)

PERMANOVA	*df*	SS	MS	*F*	*R* ^2^	*p*
Landscape (Bray–Curtis)						
Landscape type	1	0.08	0.08	2.46	0.06	.004
Site	4	0.26	0.06	2.01	0.20	.001
Residuals	30	0.97	0.03		0.74	
Total	35	1.30			1.00	
Landscape (Unifrac, unweighted)						
Landscape type	1	0.08	0.08	2.19	0.06	.042
Site	4	0.24	0.06	1.60	0.17	.042
Residuals	30	1.12	0.04		0.78	
Total	35	1.44			1.00	
Landscape (Unifrac, weighted)						
Landscape type	1	0.01	0.01	0.53	0.01	.642
Site	4	0.12	0.03	2.88	0.27	.016
Residuals	30	0.30	0.01		0.71	
Total	35	0.43			1.00	

### 3.3 Which gut bacteria differ in bees exposed to different landscapes?

To identify which gut bacterial taxa differed between bees exposed to the two different landscape types focussed on in this study, we used the test implemented in DESeq2 (Table S2, Figure 2) and the ANCOM procedure. Notably, bacterial taxa belonging to the phylum Proteobacteria (the recently described species *B. apis* (Kešnerová et al., 2016), were found to be significantly different between bees foraging on OSR farms and those from areas distant to OSR farms under both ANCOM‐ and the DESeq2‐based procedure. Specifically, one of the nine dominant species clusters of the bee gut microbiota, *B. apis*, (Kwong & Moran, 2016) was higher in relative abundance in bees exposed to agricultural landscapes distant to OSR (Distant) than bees exposed to OSR farms (OSR; Table S2, Figure 2). In contrast, taxa assigned to the same Class (Alphaproteobacteria) as *B. apis* were found at higher relative abundance in bees exposed to OSR farms using the DESeq2 test only (although we note that very few sequence reads were assigned to the Alphaproteobacteria, ≪0.05%).

Under the DESeq2 test, taxa belonging to the Acetobacteraceae, Alpha 2.1, were also found at a higher relative abundance in bees foraging on OSR. Acetobacteraceae, Alpha 2.2, were in contrast higher in relative abundance in bees foraging in agricultural landscapes distant to OSR farms, and much lower in abundance overall compared to Alpha 2.1 (Table S2). Again using DESeq2, bacteria belonging to the Lactobacillaceae Family, Phylum Firmicutes (*L. kunkeei*), known to be a dominant crop (foregut) bacteria, also common in hive materials and nectar (Corby‐Harris, Maes, et al., 2014; Kwong & Moran, 2016) were also found to be higher in relative abundance in bees exposed to agricultural landscapes distant to OSR compared to bees exposed to OSR farms. However, although included in our more stringent dataset (where taxa with <500 reads were excluded), very few sequence reads were assigned to *L. kunkeei* (≪0.05%) with the exception of one sample (~10%; LS30, Distant). In contrast, taxa belonging to the recently described bee associated species *Apibacter adventoris* were found at higher relative abundance in bees exposed to OSR farms. However, we note that similar to what was found in *L. kunkeei*,* A. adventoris* read numbers were zero or very low in most bee samples (<0.05% total reads), only a very small number of samples had read numbers in the hundreds (>0.05% total reads), and one single sample had over 2,000 reads (~0.5% of total reads). Under the ANCOM test, bacteria assigned to the Lactobacillales Order (Phylum Firmicutes) were found to be higher in bees exposed to OSR farms. However, again read numbers in this taxa were very low (under 10) in almost all bee samples and show a severely skewed distributions and a few outliers.

## 4 DISCUSSION

In this study, we investigate the association between the gut microbiome and environmental landscape. We find that bees exposed to different landscape types and apiary sites exhibit significant differences in their gut microbial communities, although the variance explained by landscape type is relatively low. Specifically, we find that some taxa belonging to dominant members of the bee gut microbiota are differentially represented in bees foraging on the mass‐flowering crop oilseed rape, compared to those not foraging on this crop.

Our results lend further support to the presence of a core gut microbial community in honey bees with the main taxa previously characterized also being found in our samples (Moran, 2015). High consistency in the honey bee microbiome suggests that mutualistic relationships exist between the host and at least some members of the community, and comparative analysis of gene contents conducted in previous studies suggest beneficial roles in nutrition and digestion, while experiments with bumblebees have shown gut bacterial taxa offer protection from pathogens (Engel et al., 2012; Koch & Schmid‐Hempel, 2011b, 2012; Martinson et al., 2011; Moran, 2015). This may then imply that factors causing deviations from the normal microbial community in social bees are detrimental.

### 4.1 Microbial association with landscape exposure

We found that some members of the dominant microbial community of honey bee workers differ in relative abundance according to landscape exposure. These results are concordant across two different metrics (Bray–Curtis dissimilarities, Unifrac unweighted distances). We also find no difference in microbial community diversity (Shannon diversity index) between landscape types. Further, we also find differences in honey bee microbial diversity depending on site differences. Overall these results suggest that the environment that bees are exposed to, including environmental differences between individual sites, may affect their microbial community, particularly the relative abundance of some key taxa.

Specifically, here, we focus on reporting taxa found to be different in abundance between bees foraging on landscape types where landscape differences are known (i.e. OSR vs. not foraging on OSR), rather than between individual sites. Both tests of abundance we used show that honey bee workers foraging on OSR farms had a lower relative abundance of a dominant member of the bee gut community, an Alphaproteobacteria species *B. apis*, than bees not foraging on OSR. *Bartonella apis* shares >95% 16S rRNA sequence similarity with other species of the genus *Bartonella* which are a group of mammalian pathogens transmitted by bloodsucking arthropods (Kešnerová et al., 2016). Further, potentially key in the context of different environments, it has recently been shown that *B. apis* encodes genes which may be involved in the degradation of secondary plant metabolites (Segers et al. 2017). By contrast, taxa assigned to the same Class as *B. apis* (Alphaproteobacteria) were higher in relative abundance in bees foraging on OSR than those not foraging on OSR (supported singly by DESeq2 and therefore reported more cautiously). Also under the DESeq2 analysis only, taxa assigned to the Acetobacteraceae, Alpha 2.1, (also Class Alphaproteobacteria) were also found at higher relative abundances in bees foraging on OSR. The Alpha 2.1 group are predominantly found in the adult gut of several bee species, but also in nectar, pollen, hive materials, and larvae (reviewed in (Kwong & Moran, 2016). Alpha 2.2 (Acetobacteraceae) on the other hand were found in higher relative abundance in bees exposed to agricultural landscapes distant to OSR farms. Unsurprisingly, Alpha 2.2 were low in abundance overall in the foraging worker gut communities studied here as this taxa has been designated as a core hive bacterium that is specific to bees that feed the brood with royal jelly secreted from nurse hypopharyngeal glands (Corby‐Harris, Synder, et al., 2014). This taxa has also been reported to have a positive effect on honey bee larval survival (Corby‐Harris, Synder, et al., 2014), thus in future it may be interesting to determine whether the trend observed here in foragers is reflected in nurse workers and the hive environment.

Using DESeq2, we also find different abundances under the different environments of the Firmicutes bacteria, *L. kunkeei*, a dominant crop (foregut) species rare in the gut, but also common in materials in the honey bee environment, and *A. adventoris*. The crop microbial environment has been suggested to be functional in inoculation and decontamination of food resources (Corby‐Harris, Maes, et al., 2014), however trends observed here in *L. kunkeei* and also *A. adventoris* require further investigation as these species were represented in very low read numbers, there was large read number variation in some samples, and we did not include the crop in our analysis of the gut.

What do such differences in a bee's microbial community mean? To date, the most abundant gene function category (Clusters of Orthologous Groups – COG) found to be enriched in the honey bee metagenome, in a metagenome sequencing study (Engel et al., 2012), is the category carbohydrate metabolism and transport (20%). A further carbohydrate‐related function enriched in the honey bee, and detected across all major gut bacterial taxa, is the family arabinose efflux permease with many of these proteins showing homology to drug resistance efflux pumps (Engel et al., 2012). Importantly, these efflux pump functions may therefore be further selected upon when honey bees are exposed to pesticides or antimicrobial compounds (Engel et al., 2012). Interestingly, a recent study of the gut microbiome of bees exposed to commonly used in‐hive pesticides (coumaphos, *tau*‐fluvalinate, and chlorothalonil) found that pesticide exposure affected the impact of environment site on the honey bee bacterial community (Kakumanu, Reeves, Anderson, Rodrigues, & Williams, 2016). Furthermore, it has been shown in *Riptortus pedestris* stinkbugs that gut bacterial symbionts confer resistance to chemical insecticides (fenitrothion), with exposure to the insecticide enriching the insecticide‐degrading bacteria (*Burkholderia*) in the agroecosystem, that are then environmentally acquired by insect hosts (Kikuchi et al., 2012). We note that the concentrations of neonicotinoid pesticides detected in our studies are on the low end of the scale compared to those reported in other studies, and 15 times lower than those reported by Rundlöf et al., (2015) where negative impacts on colony growth and reproduction were found for bumblebee colonies, but not honey bee colonies (see also Balfour et al., 2017). There are a number of reasons for these differences including the fact that neonicotinoids are readily leached from seed dressings leaving a variable amount of the active ingredient to be absorbed by the plant's root system, and that winter (the OSR farm sites in our study were “winter‐sown” crops) is the season where the maximum transport of agrochemical pollutants to watercourses occurs (Sur & Stork, 2003; Wilson, Ball, & Hinton, 1999). However, interestingly, Balfour et al. (2017) showed that under the low neonicotinoid concentrations, there was a small but significant negative relationship between pollen and honey contamination, and colony weight gain.

Additionally, pollen from different plant species differs in the composition of secondary metabolites such as polyphenols and other aromatic compounds. In a recent comparative genomics study of *Bartonella,* it was shown that *B. apis* possess pathways for the degradation of aromatic compounds and that these pathways may facilitate the breakdown of specific pollen components (Segers et al. 2017). It is therefore plausible that some of the differences found here between gut bacterial communities of bees exposed to different landscapes, and between individual apiary sites, relate to differences in diet and pesticide exposure, however direct experimental tests are required to confirm this. Also noteworthy, is that the taxa represented in different relative abundances in the different landscape types in the current study do not overlap with taxa suggested to trend toward increased prevalence and diversity in more productive colonies (e.g. *Lactobacillus* species such as Firm‐4) (Horton, Oliver, & Newton, 2015). Interestingly, Horton et al., (2015) also suggest that overall colony productivity is not consistently correlated with forager gut microbial community.

Mass‐flowering crops such as OSR have been shown to enhance pollinator abundance because they provide additional pollen and nectar resources (Holzschuh, Dormann, Tscharntke, & Steffan‐Dewenter, 2013; Riedinger, Renner, Rundlöf, Steffan‐Dewenter, & Holzschuh, 2014; Schürch, Couvillon, & Ratnieks, 2016; Westphal, Steffan‐Dewenter, & Tscharntke, 2003). Therefore, greater abundance of forage and of a specific forage type may drive microbial community composition. The nutritional quality of pollen, also including the alternative pollen resources detected for different sites, may also affect community composition. For example, if bees need to consume more pollen to acquire sufficient nutrients, more pollen may potentially accumulate in the rectum and in turn more bacteria may be able to colonize the rectum. In addition, higher stress levels have been found to cause a reduction in microbial community diversity in other systems (Stothart et al., 2016) and could potentially cause a reduction in the ability of worker bees to combat infections. Honey bees exposed to the neonicotinoid imidacloprid, for example, have been reported to show an increase in infection of *Nosema* spp gut parasites (Pettis, vanEngelsdorp, Johnson, & Dively, 2012). We found no difference in microbial community diversity between the different environments, but it is important to note that the interaction between infection and microbial community is complex and can operate in both directions.

To date, host environmental habitat and the ecological conditions shaping the microbial community in the field (as opposed to lab reared hosts) has received comparatively little attention. However, habitat type (seminatural vs. cranberry farm agricultural sites in the USA) was found to have little effect on bumblebee gut microbiota (Cariveau, Powell, Koch, Winfree, & Moran, 2014). By comparison, in a recent characterization of a large number of insects and their associated gut bacteria, relative bacterial abundances in the gut were found to vary according to the environmental habitats of the insects (Yun et al., 2014). This variation was suggested to be most likely associated with the levels of oxygen available in the habitat of the insects (Yun et al., 2014).

We provide evidence for some influence of environmental exposure, broad landscape type, and also different individual apiary sites, on honey bee microbial community. Our results underscore the possibility that different landscape parameters, such as forage type and neonicotinoid pesticide exposure, may influence dominant honey bee gut bacteria and that future laboratory‐based studies are imperative for understanding what is driving these differences. This work highlights the complex interplay of the host, its gut bacteria, and the environment, and identifies focal bacteria taxa as targets for functional analyses.

## CONFLICT OF INTEREST

None declared.

## AUTHOR CONTRIBUTION

JCJ conducted study design, fieldwork, laboratory work, analyses, and wrote the paper. CF conducted analyses and wrote the paper. FH conducted analyses and wrote the paper. HAT conducted fieldwork and analyses. NJB conducted fieldwork and analyses. PB contributed to writing and editing the paper. PE developed the custom honey bee bacterial database and contributed to writing the paper. FLWR conducted study design. WOHH conducted study design and wrote the paper.
