# Supplementary material for: Gut microbiota composition is associated with environmental landscape in honey bees
Source: Ecol Evol. 2017 Nov 30;8(1):441–51. doi: 10.1002/ece3.3597 (PMC5756847; doi:10.1002/ece3.3597)
Supplement: Supplementary file 3 [file ECE3-8-441-s003.docx]

**Supporting Information**

**Table S1.** Summary of sampling design in landscape level environmental exposure experiment.

**Table S2**. Results based on pairwise comparisons of OTU representation between honey bees exposed to different landscape types (test implemented in DESeq2, mean = mean of normalized counts for all samples, false discovery rate controlled for using the Benjamini and Hochberg procedure (Benjamini & Hochberg 1995)).
